# Multiple-Electrode Switching-Based Radiofrequency Ablation vs. Conventional Radiofrequency Ablation for Single Early-Stage Hepatocellular Carcinoma Ranging From 2 to 5 Cm

**DOI:** 10.3389/fonc.2020.01150

**Published:** 2020-07-24

**Authors:** Guang-liang Huang, Ming Liu, Xiao-er Zhang, Bao-xian Liu, Ming Xu, Man-xia Lin, Ming Kuang, Ming-de Lu, Xiao-yan Xie

**Affiliations:** ^1^Department of Medical Ultrasonics, Institute for Diagnostic and Interventional Ultrasound, The First Affiliated Hospital of Sun Yat-sen University, Guangzhou, China; ^2^Department of Liver Surgery, The First Affiliated Hospital of Sun Yat-sen University, Guangzhou, China

**Keywords:** radiofrequency ablation, hepatocellular carcinoma, multiple-electrode switching, local tumor progression, treatment outcome

## Abstract

**Purpose:** To retrospectively compare the treatment outcome of multiple-electrode switching-based radiofrequency ablation (switching RFA) and the conventional RFA for early-stage hepatocellular carcinoma (HCC).

**Methods:** A total of 122 patients with single early-stage HCC ranging from 2.1 to 5.0 cm received ultrasonography-guided percutaneous RFA as the first-line treatment. Seventy-one patients underwent switching RFA, and 51 underwent conventional RFA. Tumor response, major complication, local tumor progression (LTP), and overall survival (OS) were compared between the two groups. Log-rank tests and Cox regression models were used for univariate and multivariate analyses to identify predictors of LTP and OS.

**Results:** The rate of initial local complete response rates were 100% (71/71) in the switching RFA group and 98.0% (50/51) in the conventional RFA group (*P* > 0.05). No major complication occurred in the switching RFA group, whereas two in the conventional RFA group. After a median follow-up period of 45.9 months (range, 9.8–60.0 months), the rates of LTP in the switching RFA and conventional RFA groups were 19.7% (14/71) and 41.2% (21/51), respectively. The cumulative LTP rates at 1, 3, and 5 years were 11.3, 20.5, and 20.5% for switching RFA and 17.6, 38.7, and 46.7% for conventional RFA, respectively (*p* < 0.001). Switching RFA was an independent factor associated with a lower LTP rate (*p* = 0.022). Five-year OS rates were 75.8% after switching RFA vs. 66.2% after conventional RFA (*p* = 0.363). Extrahepatic recurrence was a significant prognostic factor for OS in multivariable analysis.

**Conclusion:** Compared with conventional RFA, switching RFA provides a high local tumor control for single early-stage HCC. An ongoing randomized trial might help to clarify the role of this approach for the treatment of HCC.

## Introduction

Radiofrequency ablation (RFA) has been widely used in the treatment of early-stage hepatocellular carcinoma (HCC) (1–4). Compared with surgical resection, the higher local tumor progression (LTP) rate is regarded as a considerable shortcoming of RFA in the treatment of HCC (5, 6). Because of the limited coagulated necrosis induced by RFA, the ability of local control of HCC with RFA greatly depends on tumor size (7–9). To achieve complete ablation of HCC and sufficient safety margin, overlapping ablations are required (10–12). For conventional RFA with one electrode, the electrode is repositioned and reactivated in untreated tumor sites adjacent to the prior ablation zone after each ablation. The hyperechogenicity caused by the early radiofrequency (RF) electrode frequently obscures the tumor boundaries, rendering the reposition of the electrode under ultrasound (US) guidance, which is technically challenging and time consuming even for a relatively small tumor. A multiple-electrode switching system was introduced and enabled to simultaneously use up to three RF electrodes, between which the power is sequentially switched when an impedance spike is encountered, instead of temporarily switching the system off as it occurs in the conventional RF device (13, 14). The problem mentioned earlier can be potentially remedied with the introduction of such a multiple-electrode switching system. Several previous reports have shown the encouraging results of multiple-electrode switching-based RFA for the treatment of HCC. However, all these studies were single-arm studies without direct comparison with conventional RFA (15–17). The aim of this study was thus to retrospectively compare the treatment outcome of multiple-electrode switching-based RFA (switching RFA) and the conventional RFA with a single electrode for early-stage HCC.

## Patients and Methods

### Patients

**Figure 1:**
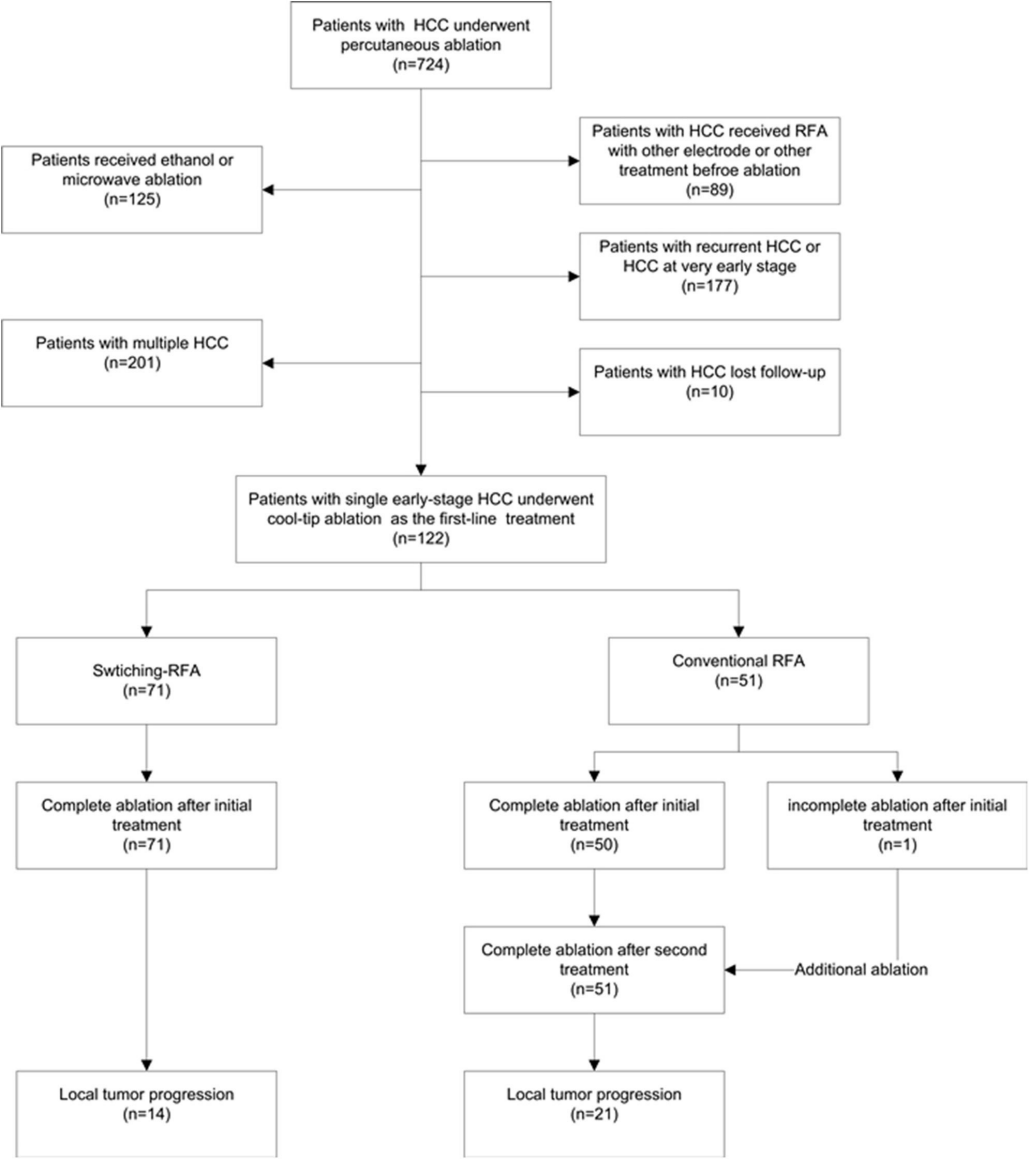
Patient flow diagram for analyses.

From August 2009 to August 2014, a total of 122 patients (105 men, 17 women; mean age 56.3 ± 12.6 years, range: 27.0–85.0 years) with HCC were enrolled in this retrospective study (Figure 1). The diagnosis of HCC was based on the noninvasive diagnostic criteria of the American Association for the Study of Liver Diseases (AASLD) or biopsy. The inclusion criteria comprised: (a) adult patients with early-stage HCC and declined resection recommendation; (b) single HCC with 2.1–5.0 cm in diameter, treated by conventional cool-tip electrode RFA system or multiple-electrode switching RFA system; (c) liver function status at Child–Pugh class A or B; (d) platelet count over 50 × 10^9^/l; and (e) prothrombin time ratio >50%. Exclusion criteria include (a) presence of multiple HCCs; (b) presence of vascular invasion or extrahepatic metastases at pre-procedure imaging study; (c) previous treatment for HCC; (d) ongoing anticoagulant treatment that cannot be stopped; and (e) tumor in close proximity to the hepatic hilum. The study was conducted with the approval of the institutional ethics board. Written informed consent was obtained from each patient before treatment.

### Radiofrequency Ablation

US-guided percutaneous RFA was performed under local anesthesia and sedation. Vital signs were continuously monitored during the procedure. Two of the authors (X.Y. X. and M. K., who have 10 and 8 years of experience with RFA, respectively) performed the ablation. RFA was carried out with the Cool-tip^TM^ system (Valleylab, Boulder, CO, USA). For conventional RFA, a 17-gauge internally cooled electrode with a 2- to 3-cm-long exposed metallic tip was used. Grounding was achieved by attaching a dispersive pad to each of the patient's thighs. Overlapping ablation technique was used, and the generator was set at the maximum power of 200 W in the impedance automatic mode for 8–12 min. For switching RFA, two or three internally cooled electrodes (Covidien, Boulder, CO) were used, and two grounding pads were placed on each of the patient's thighs. The generator was set at the maximum power of 200 W in the impedance switching mode for 16–24 min. The selection of electrode number and the length of exposed electrode tip was primarily determined based on tumor size and tumor location. Generally, two RF electrodes were used for tumors 2.1–3.0 cm, and three for tumors 3.1–5.0 cm in diameter, with an interelectrode distance of 1.0–2.0 cm. Regardless of the ablation with or without a multiple-electrode switching system, the needle track was carefully treated with the electrode being retracted by a 1-cm increment to prevent bleeding and tumor seeding. Contrast-enhanced US was performed immediately after the RFA procedure in order to obtain complete tumor ablation and a 5-mm safety margin as far as possible.

### Evaluation of Treatment Response and Follow-Up

Local efficacy was assessed by a conventional evaluation modality of contrast-enhanced CT or MRI performed 1 month after ablation. According to the CT/MRI results, a response to RFA was classified as complete or incomplete ablation. Complete ablation was defined as non-enhancement in the ablated zone with or without peripheral enhancing rim. Incomplete ablation was indicated when the tissue was still enhanced at the tumor site, and additional ablation was given. If the tumor was still viable after additional ablation, RFA was considered a failure, and the patient was referred for other therapies. The follow-up protocol included contrast-enhanced US performed at 3-month intervals and contrast-enhanced CT performed every 6 months. LTP was defined as the regrowth of the tumor inside the initially completely ablated nodule. All the ablation-related complications were classified according to the Society of Interventional Radiology Reporting Standards for image-guided tumor ablation (18). The follow-up duration was defined as the interval between the first RF ablation and either the incidence of the event or the last visit before December 31, 2018. The follow-up for survival analysis was terminated at 60 months. Liver transplantation was censored on the date of surgery.

### Statistical Analysis

Statistical analysis was performed using the SPSS 16.0 software package. A *p*-value of <0.05 indicated statistical significance. Continuous data were expressed as the mean ± standard deviation. The chi-squared test or Fisher exact tests were used to compare patients' baseline characteristics. Data on survival were evaluated by the Kaplan–Meier method. The relationship between each of the variables and LTP or overall survival (OS) was estimated by the log-rank test. The variables included age, sex, presence of hepatitis B or C virus infection, Child–Pugh class for liver function, alanine aminotransferase, total bilirubin, albumin, prothrombin time, platelet, serum alpha-fetoprotein, tumor location (perivascular or subcapsular), tumor size, and treatment methods. The variables with a *p*-value of <0.10 in the log-rank test were introduced in a multivariate Cox proportional hazards model. Perivascular HCC was defined as an index tumor abutting the first- or second-degree branches of a portal or hepatic vein that are 3 mm or greater in diameter. Subcapsular tumor was defined as an index tumor being <1.0 cm from the liver capsule.

## Results

### Patients

Of the 122 eligible patients, 71 (mean age, 55.5 ± 12.2 years; range, 27–80 years) underwent switching RFA, whereas 51 (mean age, 57.4 ± 13.1 years; range, 27–80 years) underwent conventional RFA. The tumor sizes were 2.8 ± 0.5 cm (range, 2.1–4.4 cm) in the switching RFA group and 2.8 ± 0.7 cm (range, 2.1–4.6 cm) in the conventional RFA group. Among 26 perivascular tumors, 18 (69.2%) tumors were abutting portal vein, 6 (23.1%) abutting hepatic vein, and 2 (7.6%) abutting inferior vena cava. The baseline clinical characteristics of the two groups are compared in Table 1. No significant differences were observed between the two groups in sex, age, Child–Pugh class, presence of hepatitis B/C virus infection, serum total bilirubin, serum albumin, tumor size, and tumor location (perivascular or subcapsular tumor), whereas the serum alanine aminotransferase level, prothrombin time, platelet count, and the serum alpha-fetoprotein level differed significantly.

**Table 1:** Baseline characteristics of patients with single early-stage HCC.

**Characteristics**	**Switching-RFA**	**Conventional RFA**	***P*-value**
	**(*n* = 71)**	**(*n* = 51)**	
Gender (male/female)	64/7	41/10	0.125
**Age (y)**			
≤65	58	36	0.150
>65	13	15	
Child-Pugh (A/B)	69/2	48/3	0.704
Hepatitis B/C (±)	67/4	48/3	1.000
**Serum ALT level**			
≤40 U/L	46	22	0.018
>40 U/L	25	29	
**Serum total bilirubin**			
≤34.2 mg/dl	65	49	0.531
>34.2 mg/dl	6	2	
**Serum albumin**			
≤35 g/L	13	16	0.095
>35 g/L	58	35	
**Prothrombin time**			
≤14 s	64	37	0.011
>14 s	7	14	
**Platelet count**			
≤100 × 10^9^/L	13	20	0.010
>100 × 10^9^/L	58	31	
**Serum AFP level**			
≤200 ng/ml	64	36	0.006
>200 ng/ml	7	15	
**Tumor size**			
2.1–3.0 cm	50	35	0.832
3.1–5.0 cm	21	16	
Perivascular (+/–)	18/53	8/43	0.198
Subcapsular (+/–)	21/50	22/29	0.122

### Tumor Response

**Figure 2:**
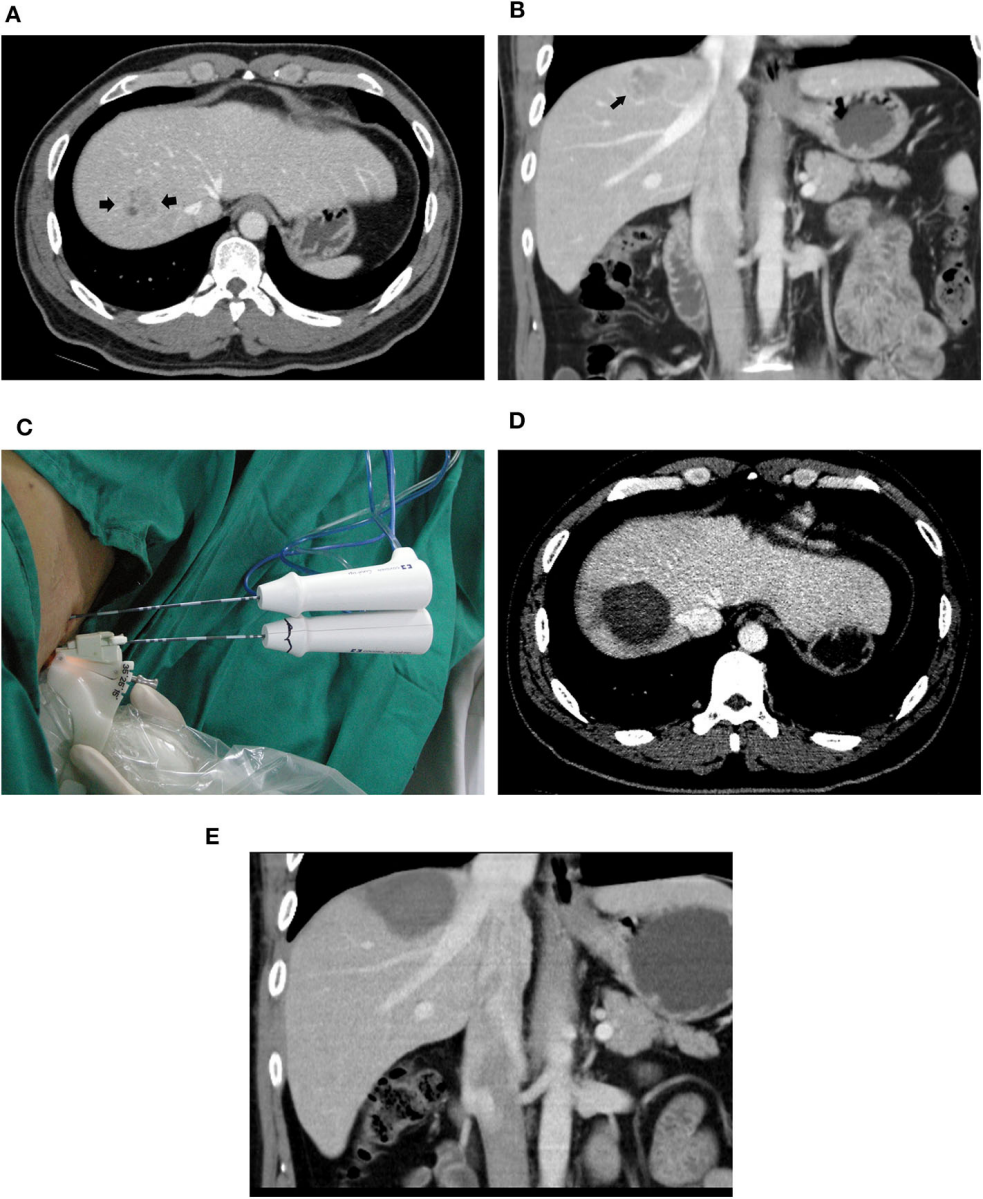
A 39-year-old male patient with HCC who underwent radiofrequency ablation with a multiple-electrode switching system. Pre-ablation CT scans showed a 2.6-cm tumor (arrowhead) in the segment VIII of the liver in the transverse **(A)** and coronal **(B)** view. **(C)** Ultrasound-guided RFA using the multiple-electrode switching system was performed with two RF electrodes for 16 min. CT scans obtained 1 month after ablation showed that the tumor was completely ablated in the transverse **(D)** and coronal **(E)** view.

In the switching RFA group, complete ablation was achieved in all tumors in a single session of RFA (Figure 2). In the conventional RFA group, complete ablation was achieved in 50 of 51 tumors in a single session of RFA. One residual tumor reached complete ablation after additional treatment. The rates of initial local complete ablation were 100% (71/71) in the switching RFA group and 98.0% (50/51) in the conventional RFA group (*P* = 0.418).

### Major Complications

No ablation-related death occurred. No major complication was observed in the switching RFA group, whereas two (2/51, 3.9%) major complications were observed in the conventional RFA group. One patient in the conventional RFA group developed obstructive jaundice as a result of the injury of the bile duct. The patient received percutaneous transhepatic catheter drainage and stent placement. Another patient required US-guided percutaneous gallbladder catheter drainage and antibiotics due to the acute cholecystitis.

### Local Tumor Progression

**Figure 3:**
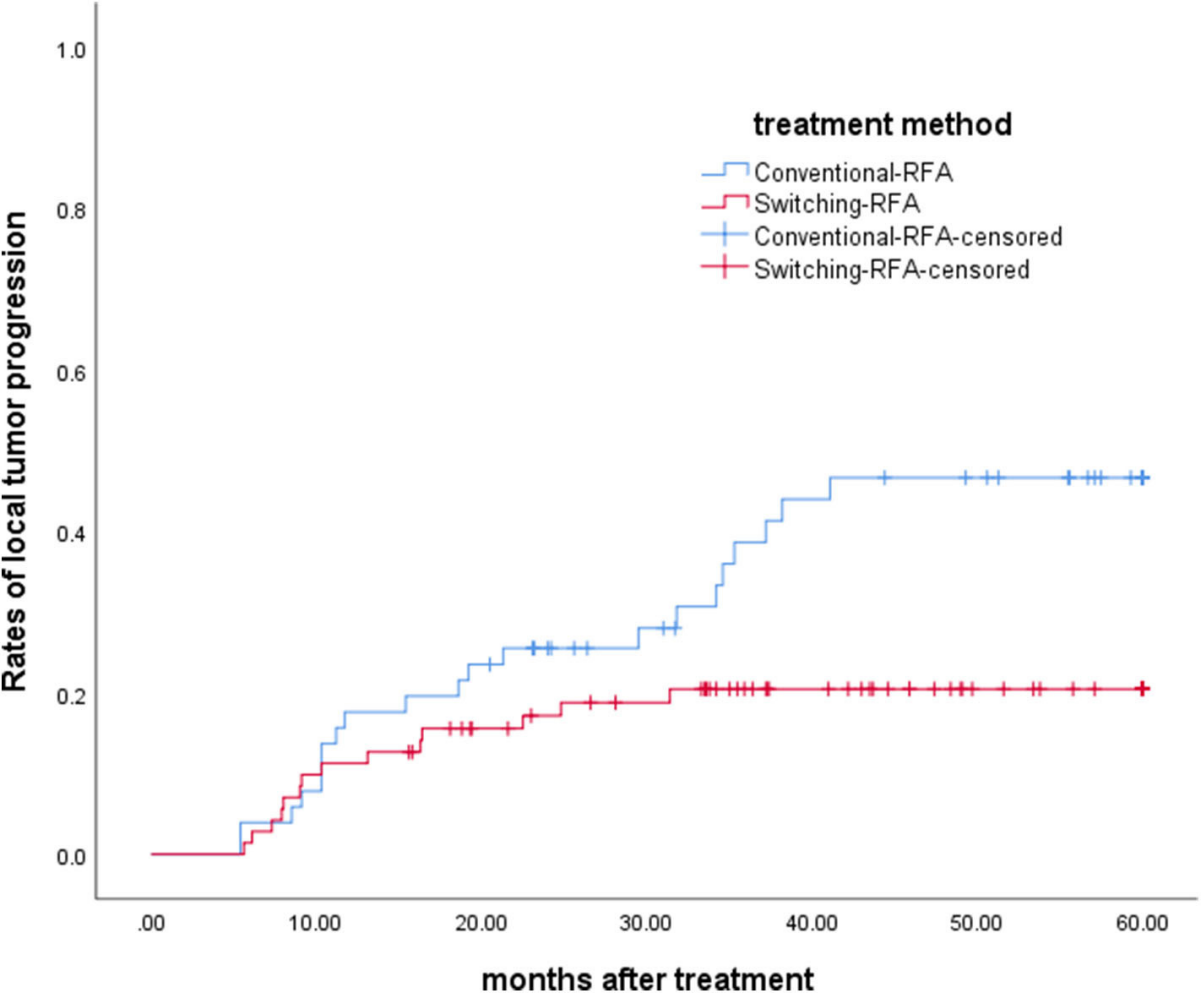
Cumulative LTP survival curves in patients treated with switching RFA or conventional RFA.

The overall median follow-up period for all patients was 45.9 months (range, 9.8–60.0 months), with median follow-up periods of 43.0 months (range, 9.8–60.0 months) and 55.3 months (range, 10.3–60.0 months) for switching RFA group and conventional RFA group, respectively (*p* = 0.107). The LTP rate in the switching RFA group was 19.7% (14/71) vs. 41.2% (21/51) in the conventional RFA group (*p* = 0.010). According to the Kaplan–Meier method, the cumulative LTP rates at 1, 3, and 5 years were 11.3, 20.5, and 20.5% for switching RFA and 17.6, 38.7, and 46.7% for conventional RFA, respectively (Figure 3). Risk factors for LTP of HCC were analyzed by the log-rank test, which revealed that the treatment method was significantly associated with LTP (*p* = 0.018). Other factors associated with LTP are described in Table 2. In a multivariable analysis, the treatment method was identified as an independent predictor of LTP (HR = 2.209; 95% CI: 1.123–4.346; *p* = 0.022) (Table 3). LTP was treated by repeat RFA (*n* = 16), ethanol ablation (*n* = 1), liver transplantation (*n* = 1), surgical resection (*n* = 2), and TACE (*n* = 1) in the conventional RFA group, whereas repeat RFA (*n* = 11), liver transplantation (*n* = 1), and surgical resection (*n* = 2) in the switching RFA group.

**Table 2:** Univariate analysis of risk factors for local tumor progression of HCC after ablation.

**Variables**	**Local tumor progression *P*-value**
Gender (male)	0.790
Age (>65 years)	0.601
Hepatitis B/C virus infection (+/–)	0.382
Child-Pugh class (A/B)	0.173
Serum ALT level (>40 U/L)	0.438
Serum total bilirubin (>34.2 mg/dl)	0.077
Serum albumin (>35 g/L)	0.089
Platelet count (>100 × 10^9^/L)	0.773
Prothrombin time (>14 s)	0.703
Serum AFP level (>200 ng/ml)	0.962
Tumor size (2.1–3.0/3.1–5.0 cm)	0.598
Perivascular (+/–)	0.129
Subcapsular (+/–)	0.665
Treatment methods (switching/non-switching)	0.018

*+, positive; –, negative; ALT, alanine aminotransferase; AFP, alpha-fetoprotein*.

**Table 3:** Multivariate analysis of risk factors for local tumor progression of HCC after ablation.

**Variables**	***P*-value**	**Risk ratio (95% CI)**
Treatment methods	0.022	2.209 (1.123–4.346)
Serum albumin	0.151	NA
Serum total bilirubin	0.096	NA

*NA, not applicable*.

### Distant Recurrence

Intrahepatic distant recurrence had occurred in 25 of 71 (35.2%) patients in the switching RFA group and in 31 of 51 (60.8%) patients in the conventional RFA group (*p* = 0.005). Extrahepatic recurrence was found in 10 of 71 (14.1%) patients in the switching RFA group and in 11 of 51 (21.6%) patients in the conventional RFA group (*p* = 0.280).

### Overall Survival Rates

Ten (14.1%) of 71 patients in the switching RFA group and 13 (25.5%) of 51 patients in the conventional group died during the observation period. The estimated 5-year OS rates were 75.8% in the switching RFA group and 66.2% in the conventional RFA group (*p* = 0.363). Of the factors evaluated for association with OS in univariate analysis (Table 4), the following three factors were statistically significant: LTP (*p* = 0.004), intrahepatic recurrence (*p* = 0.002), and extrahepatic recurrence (*p* = 0.000). Extrahepatic recurrence was found to be a significant risk factor in the Cox proportional hazards regression model (hazard ratio = 15.850; 95% CI: 6.169–40.722; *p* < 0.001; Table 5).

**Table 4:** Univariate analysis of risk factors for overall survival of HCC after ablation.

**Variables**	**Overall survival *P*-value**
Gender (male)	0.219
Age (>65 years)	0.334
Hepatitis B/C virus infection(+/–)	0.481
Child-Pugh class (A/B)	0.241
Serum ALT level (>40 U/L)	0.088
Serum total bilirubin (>34.2 umol/L)	0.151
Serum albumin (>35 g/L)	0.451
Platelet count (>100 × 10^9^/L)	0.452
Prothrombin time (>14 s)	0.689
Serum AFP level (>200 ng/ml)	0.197
Tumor size (2.1–3.0/3.1–5.0 cm)	0.798
Perivascular tumor	0.158
Subcapsular	0.114
Treatment methods (switching/non-switching)	0.363
Local tumor progression	0.004
Intrahepatic recurrence (+/–)	0.002
Extrahepatic recurrence (+/–)	0.000

*+, positive; –, negative; ALT, alanine aminotransferase; AFP, alpha-fetoprotein*.

**Table 5:** Multivariate analysis of risk factors for overall survival of HCC after ablation.

**Variables**	***P*-value**	**Risk ratio (95% CI)**
Extrahepatic recurrence	0.000	15.850 (6.169–40.722)
Local tumor progression	0.256	NA
Intrahepatic recurrence	0.337	NA
Serum ALT level	0.532	NA

*NA, not applicable*.

## Discussion

The present study demonstrated that both switching RFA and conventional RFA achieved satisfactory tumor response for single HCC, ranging from 2.1 to 5.0 cm. Switching RFA appeared to be superior in local control of HCC compared with conventional RFA, whereas similar OS was achieved after both treatments.

Previous studies have reported the LTP rate to be as high as 40% in patients with early-stage HCC, especially for the intermediate-sized HCC (19). It has been reported that HCC micrometastases may still exist as far as 1 cm from the main tumor, including the small encapsulated tumors (20). Therefore, at least 0.5 cm of the ablation margin is required for local ablative therapy (21–23). However, the commonly used RFA devices show limited ability to create a large ablation zone. To overcome the limitations of conventional RFA, other techniques, including microwave ablation (24), expandable RF electrode (24), and clustered RF electrode (12), have been proposed. In theory, Microwave ablation is more efficient than RFA and less influenced by the heat-sink effect. However, the relatively high complication rate limits its clinical use for the tumor close to the critical structures. Expandable electrodes can create a large ablation zone, but it is difficult to see all the time under US-guided RFA. Multiple-electrode switching RFA system enabled the creation of significantly larger ablation zones compared with conventional RFA system with a single-electrode *in vivo* experiment study (14). In the present study, the LTP rates at 1, 3, and 5 years were 11.3, 20.6, and 20.6% in the switching RFA group, as compared with 17.6, 38.7, and 46.7% in the conventional RFA group, respectively. Switching RFA provided a better local tumor control than conventional RFA. The result was further supported by the multivariate analysis, which highlighted switching RFA as an independent factor associated with LTP. Previous studies reported that tumor size and tumor location (subcapsular/perivascular location) were two predisposing factors to LTP of HCC (3, 25, 26). However, no such correlations were detected in our cohorts.

In the present study, no major complication was observed in the switching RFA group, whereas two major complications occurred in the conventional RFA group. As we mentioned before, the gas induced by the ablation will disturb the reposition of the electrode. Under such circumstances, we had to count the scale marks from both electrode and puncture line and tried to ensure a uniform depth in which a shift of punctate was inevitable, but it cannot be predicted. The likelihood of injuring the neighboring vital structure, such as portal branches, biliary ducts, or gastrointestinal tract, increases. Multiple electrodes in this new system can be inserted into the predetermined location, minimizing the possibility of damaging the important neighboring structure. That could be the reason why no major complication was observed in the switching RFA group in the present study. Although the rate of major complications did not differ between two groups, theoretically, patients may benefit from the multiple-electrode switching-based RFA, and we believe that it can be significant between two groups if large cohorts were analyzed, especially when more large tumors were included.

Interestingly, we found that the LTP rate and the intrahepatic recurrence rate in the switching RFA group were both lower than those in the conventional RFA group; however, no significant differences in OS were observed. Only extrahepatic recurrence was found to be a significant prognostic factor for OS in multivariable analysis. The reason could be explained that most patients with LTP or intrahepatic recurrence were eligible for rescue treatment. However, the estimated overall 5-year survival rate in the switching RFA group was higher than that in the conventional RFA group.

The main limitation of our study is its retrospective nature, which may induce selection bias. Ideally, randomized multicenter controlled clinical trials are needed to provide a complete evaluation of switching RFA for the treatment of HCC. Second, it should be made aware that the outcome of the RFA for HCC is heavily dependent on the expertise of the operators and that we focused only on patients with a single HCC, which may result in differences in the prognostic factors for OS.

In conclusion, our findings from this study demonstrate that the multiple-electrode switching-based RFA is safe and effective in treating single early-stage hepatocellular carcinoma. Compared with conventional RFA, switching RFA provides a high local tumor control for HCC. An ongoing randomized trial might help to clarify the role of this approach for the treatment of HCC.

## Data Availability Statement

All datasets generated for this study are included in the article/supplementary material.

## Ethics Statement

The studies involving human participants were reviewed and approved by Institutional Review Board approval was obtained from the First Affiliated Hospital of Sun Yat-sen University. The patients/participants provided their written informed consent to participate in this study. Written informed consent was obtained from the individual(s) for the publication of any potentially identifiable images or data included in this article.

## Author Contributions

GH and XZ accumulated the patients' data and wrote the main manuscript text. BL and MLiu analyzed the results. MX and MLin prepared all figures. MLu and MK conducted the therapy and exam. XX conceived the study, was responsible for the study planning, data analyze and manuscript preparation. All authors wrote and reviewed the main manuscript.

## Conflict of Interest

The authors declare that the research was conducted in the absence of any commercial or financial relationships that could be construed as a potential conflict of interest.
